# Supplementary material for: Potent human neutralizing antibodies against Nipah virus derived from two ancestral antibody heavy chains
Source: Nat Commun. 2024 Apr 6;15:2987. doi: 10.1038/s41467-024-47213-8 (PMC10998907; doi:10.1038/s41467-024-47213-8)
Supplement: Supplementary file 1 — Supplementary Information [file 41467_2024_47213_MOESM1_ESM.pdf]

Supplementary Information

Potent human neutralizing antibodies against Nipah virus derived from two ancestral antibody heavy chains

Chen *et al*.

Contents

Supplementary Figure 1: The binding ability and neutralization capacity of antibodies
Supplementary Figure 2: Binding curves of antibodies to henipavirus RBP alanine scan mutants
Supplementary Figure 3: The binding ability and neutralization capacity of NiV41 and NiV42 germline-reverted antibodies
Supplementary Figure 4: Efficacy of 41-6 in protection against NiV infection in hamsters
Supplementary Figure 5: Cryo-EM data processing of NiV-RBP in complex with the 41-6 Fab fragment
Supplementary Figure 6: Superimposition comparison of the NiV-RBP bound with Fab fragment or unbound, and conservation and oligosaccharide analysis of the epitope of 41-6
Supplementary Figure 7: Competition of receptor with different antibodies for binding to the RBPs
Supplementary Figure 8: Epitope comparison between antibody and receptor
Supplementary Figure 9: Comparison of NiV-RBP with the neutralizing antibodies
Supplementary Figure 10: Gating strategy for flow cytometry-based receptor blocking assay
Supplementary Table 1: Summarization of the statistics of the map reconstruction and model building

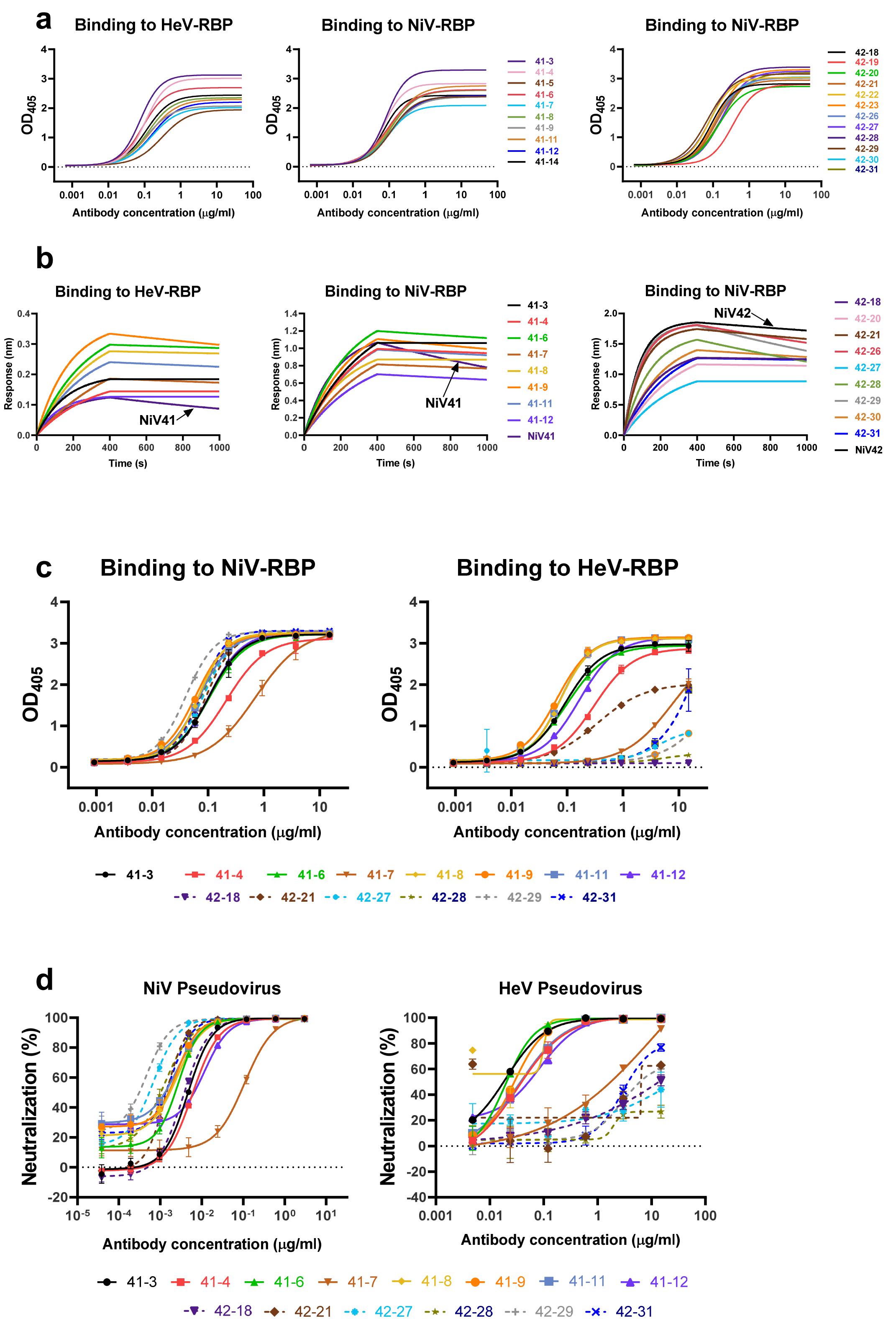

Supplementary Figure 1: The binding ability and neutralization capacity of antibodies.

a Binding curves of identified Fab clones to henipavirus RBPs. 10 Fab clones were sourced from NiV41, and the binding profiles to HeV-RBP (left) and NiV-RBP (middle) were determined by ELISA. 12 Fab clones were sourced from NiV42, and the binding

profiles NiV-RBP (right) were determined by ELISA. **b** BLI kinetic binding of identified Fab clones to henipavirus RBPs. Biotinylated RBPs were loaded onto probes. All Fabs were diluted to a final concentration of 100 nM. Arrows represent parental antibody binding. **c** Binding curves of antibodies to henipavirus RBPs. After converted to IgG format, the binding ability of mAbs to NiV (left) and HeV-RBP (right) were detected by ELISA. mAbs sourced from NiV41 and NiV42 are shown in sold lines and dashed lines, respectively. **d** Neutralization activity evolution of antibodies against NiV (left) and HeV (right) pseudoviruses. Antibodies sourced from NiV41 and NiV42 are shown in sold lines and dashed lines, respectively. Pseudoviral transduction of NiV and HeV pseudovirus was measured by GFP-positive cells and luciferase activities, respectively. Data are represented as the mean (**a**) from n=2 biologically independent experiments or mean ± S.D (**c, d**) from n=3 biologically independent experiments**.** Source data are provided as a Source Data file.

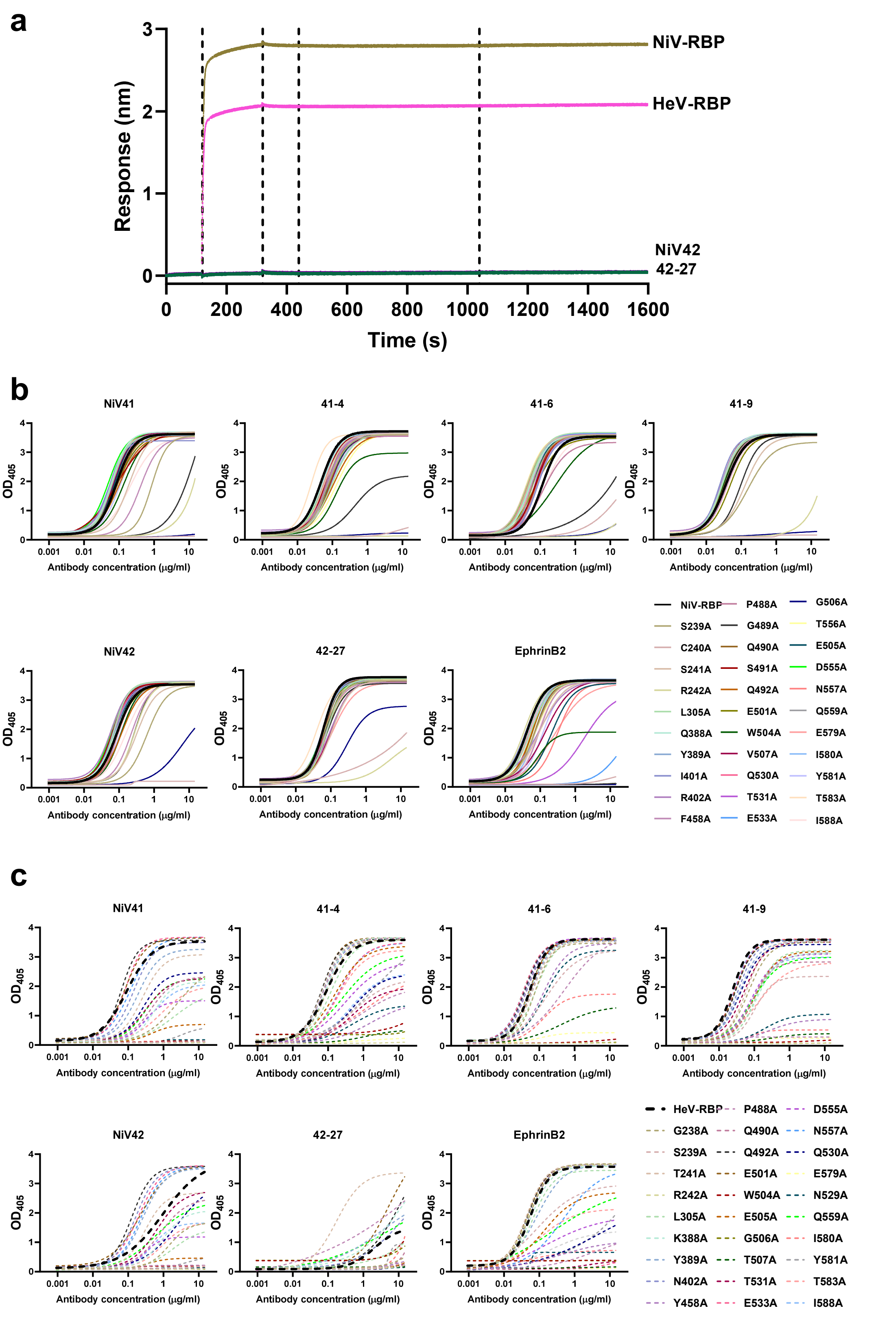

Supplementary Figure 2: Binding curves of antibodies to henipavirus RBP alanine scan mutants.

a The specificity assessment was conducted between RBPs and high concentrations of antibodies interacting with the probe. The biotinylated RBPs were immobilized onto SA

probes at a concentration of 35 μg/ml. The interaction with the probes was then monitored during both the association and dissociation phases. Similarly, the Fab fragments of NiV42 and 42-27, at a concentration of 2000 nM, were used to detect the reaction to the probes. **b** Binding ability of antibodies to NiV-RBP alanine scan mutants. By analyzing the structure of the receptor protein and the RBP complex (PDB 2VSM), 32 key amino acids were selected for mutation and expressed as recombinant proteins. the binding ability of antibodies to mutants were detected by ELISA. The binding results was analyzed by fitting to four–parameter curve using Graphpad Prism software. Wild-type antigen was highlighted as thick curve. **c** Binding ability of antibodies to HeV-RBP alanine scan mutants. By analyzing the structure of the receptor protein and the RBP complex (PDB 6PDL), 29 key amino acids were selected for mutation and expressed as recombinant proteins. the binding ability of antibodies to mutants were detected by ELISA. The binding results was analyzed by fitting to four–parameter curve using Graphpad Prism software. Wild-type antigen was highlighted as thick dashed curve. Data are represented as mean values of duplicates. The experiments were done once with technical duplicates. Source data are provided as a Source Data file.

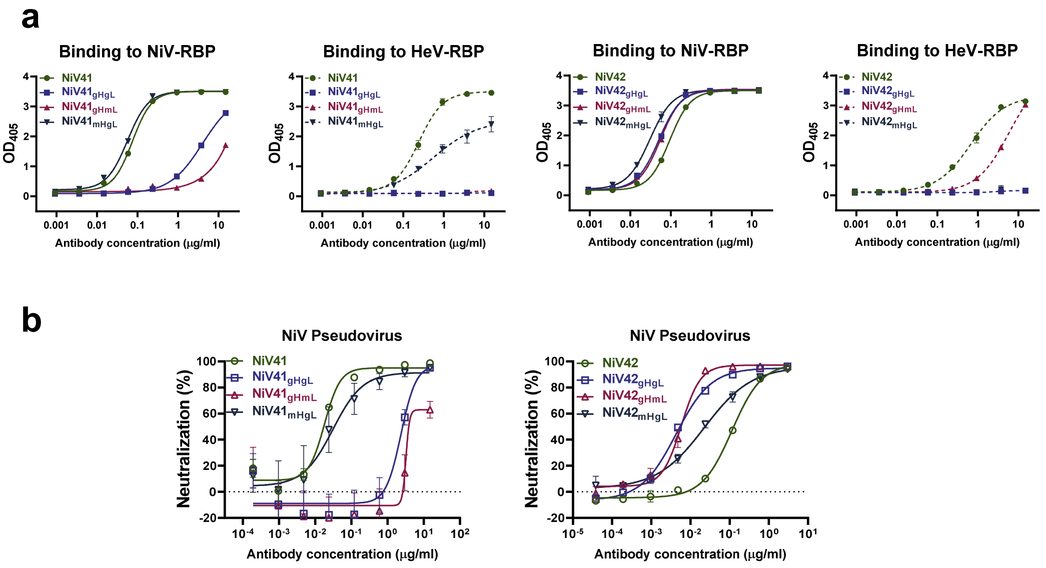

Supplementary Figure 3: The binding ability and neutralization capacity of NiV41 and NiV42 germline-reverted antibodies.

a Binding curve of germline-reverted antibodies to RBPs. **b** Neutralization activity evolution of germline-reverted antibodies against NiV pseudovirus. gHgL, VH germline paired with VL germline. gHmL, VH germline paired with VL germline. mHgL, antibody VH paired with VL germline. Data are represented as the mean ± S.D from n=3 biologically independent experiments**.** Source data are provided as a Source Data file.

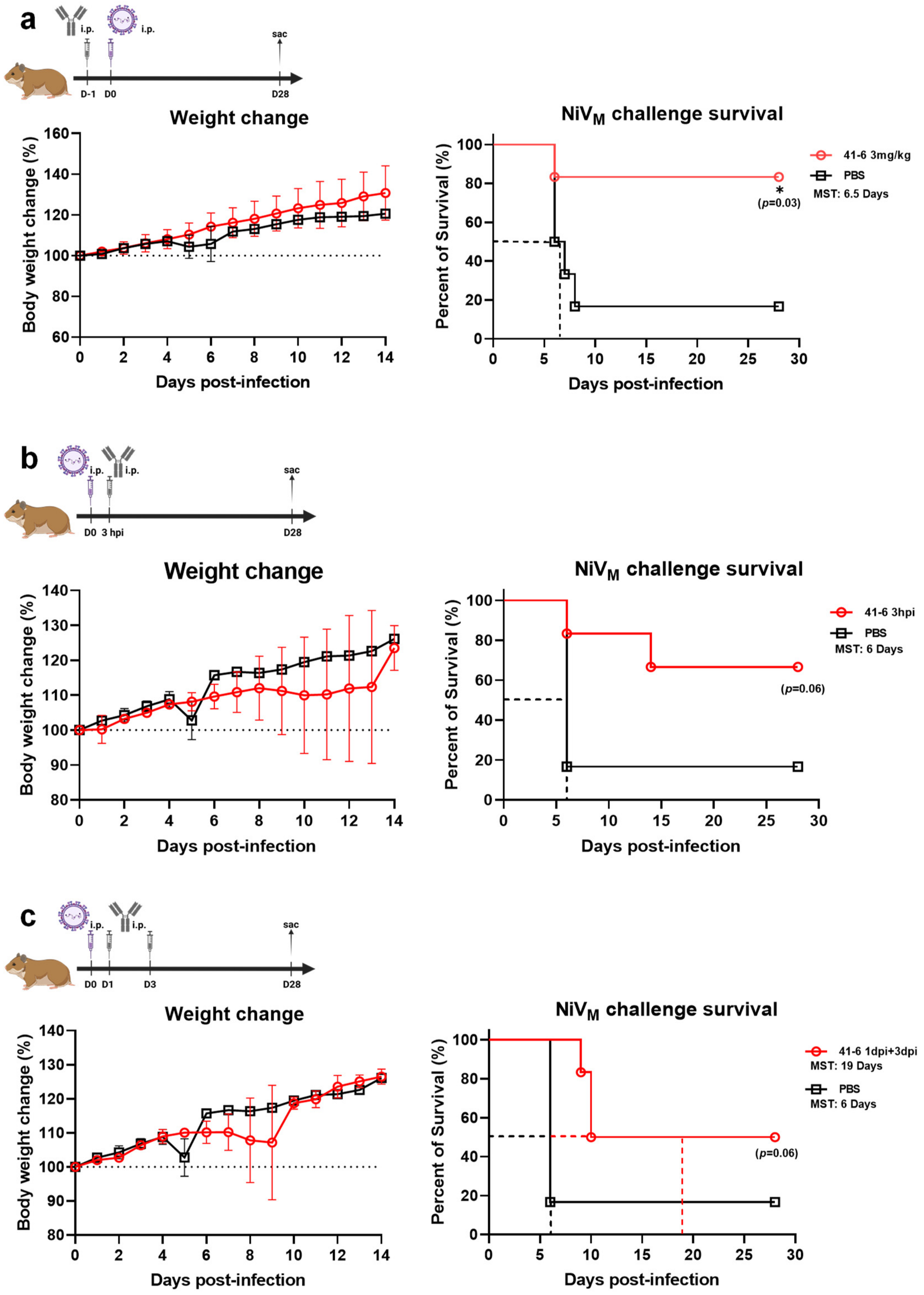

Supplementary Figure 4: Efficacy of 41-6 in protection against NiV infection in hamsters.

a Prophylactic efficacy of 41-6 against a lethal challenge with the NiV Malaysia strain. Hamsters were treated with 3 mg/kg 41-6 or PBS 24 hours before intraperitoneal inoculation with NiV virus. **b** Therapeutic efficacy of 41-6 against a lethal challenge with the NiV Malaysia strain. Hamsters were treated with 10 mg/kg 41-6 or PBS 3 hours after intraperitoneal inoculation with NiV virus. **c** Therapeutic efficacy of 41-6 against a lethal

challenge with NiV Malaysia strain. Hamster were treated with PBS buffer or 10 mg/kg 41-6 1 day and 3 days after intranasal inoculation with NiV virus. The weight change was collected daily for 14 days after inoculation and the survival was observed for a duration of 28 days. Error bars represent the mean ± S.D. Data (n=6 biologically independent animals) were analyzed by the two-sided Log-rank (Mantel–Cox) test using Prism software (*p<0.1, ***p<0.001)**.** MST, median survival time. The schemes were created with BioRender.com. The PBS-treated animals in **b, c** and Fig. 5b are identical. Source data are provided as a Source Data file.

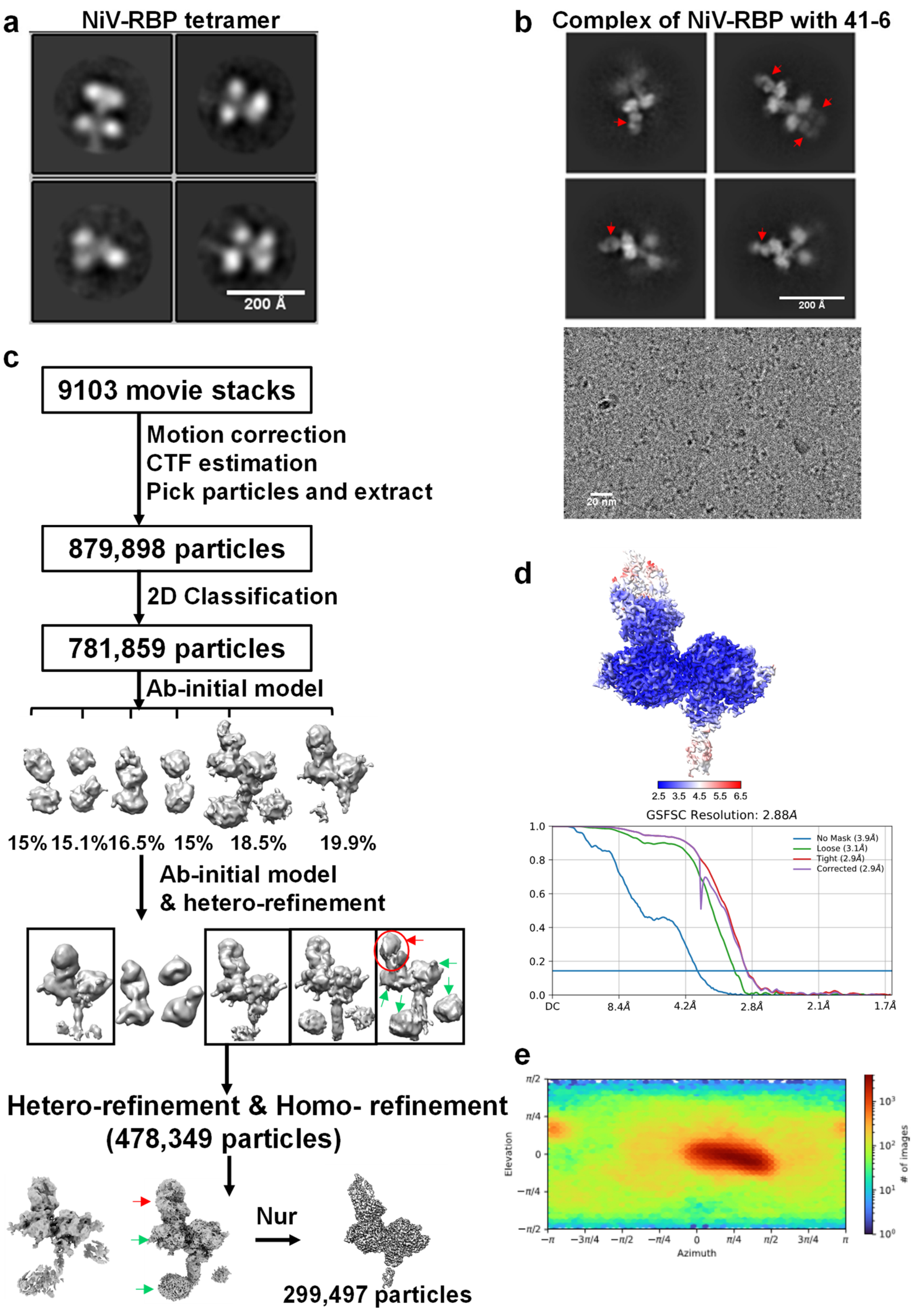

Supplementary Figure 5: Cryo-EM data processing of NiV-RBP in complex with the 41-6 Fab fragment.

a Verification of the structural correctness of the NiV-RBP ectodomain using negative-stain EM. **b** Representative motion-corrected Cryo-EM micrographs and reference-free 2D class averages of NiV-RBP with 41-6 complex. Red arrows indicate Fab. **c** Workflow

of the data processing. Green arrows indicate NiV-RBP head domain, Red arrows indicate Fab. **d** Gold standard FSC plots and local resolution map for the 3D reconstruction, calculated in cryoSPARC. **e** Euler angle distribution of the particle images.

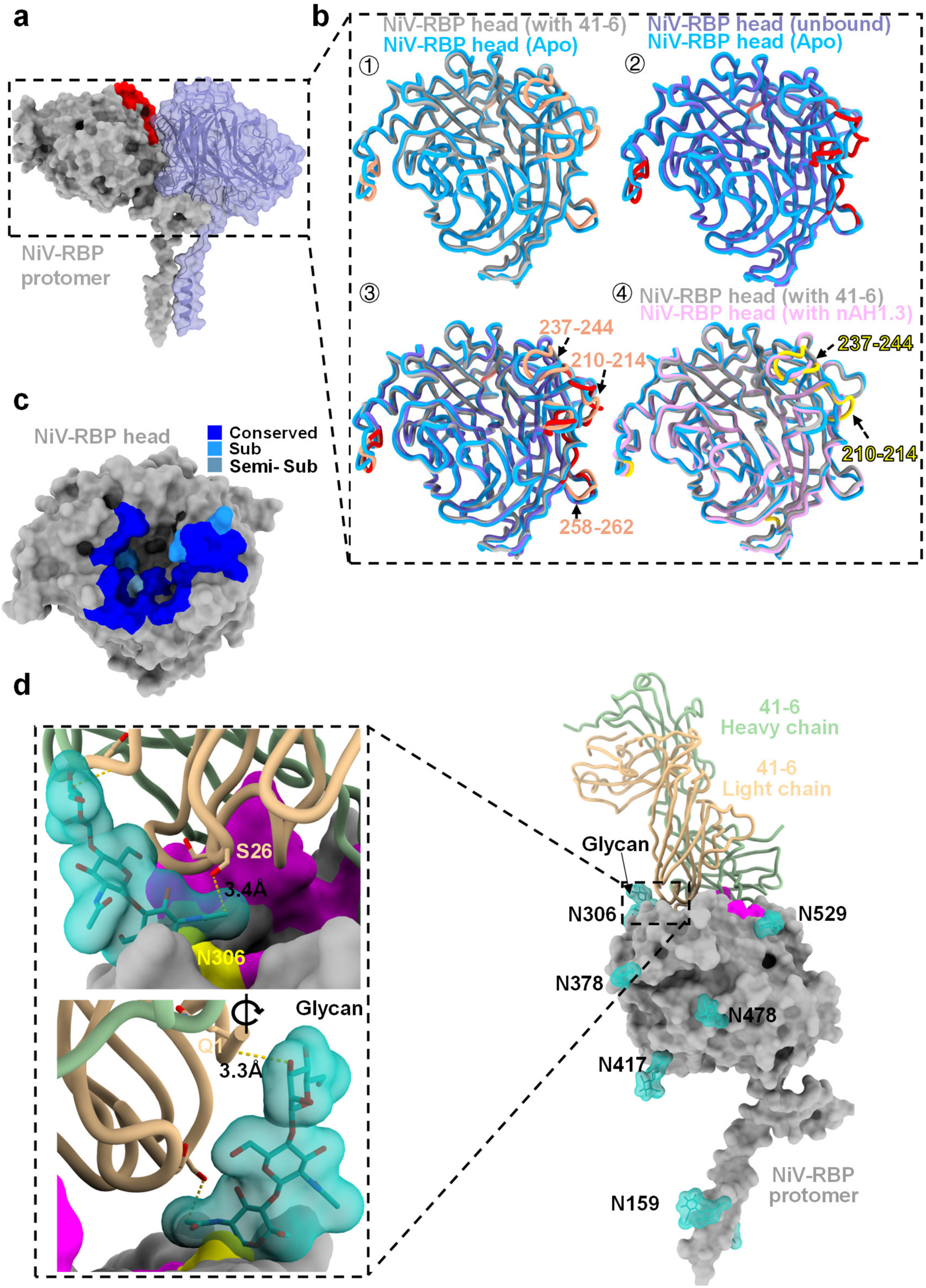

Supplementary Figure 6: Superimposition comparison of the NiV-RBP bound with Fab fragment or unbound, and conservation and oligosaccharide analysis of the epitope of 41-6.

a Left shown as molecular surface, **b** Right zoomed-in view of head structure of comparison NiV-RBP tetramer bound with 41-6 (①③) or unbound (②③) to apo NiV-RBP (PDB 3D11) and NiV-RBP bound with 41-6 to bound with nAH1.3 (④) (PDB 7TXZ). Red, Salmon and Gold highlights show respectively regions of Cα trajectory with the deviations

(3 Å ＞RMSD＞1.5 Å) between NiV-RBP tetramer head without 41-6 and apo NiV-RBP, NiV-RBP tetramer head with 41-6 and apo NiV-RBP, NiV-RBP tetramer head with 41-6 and NiV-RBP with nAH1.3. **c** Conserve epitopes of henipavirus RBP bound with 41-6 superimposed. Molecular surface representation of the NiV-RBP bound with 41-6. Sub, conservative substitution; semi- sub, semiconservative substitution. **d** The impact of N-glycan docked on NiV-RBP bound with 41-6. NiV-RBP is shown as a molecular surface, and 41-6 are shown as ribbon diagrams. Upper and lower left, two magnified views of the distance between N-glycan and ASN306 (N306) close to 41-6. Dark turquoise indicates N-glycan (from PDB 7YT0), and the distance between N-linked glycosylation at position N306 is shown as a gold dotted line.

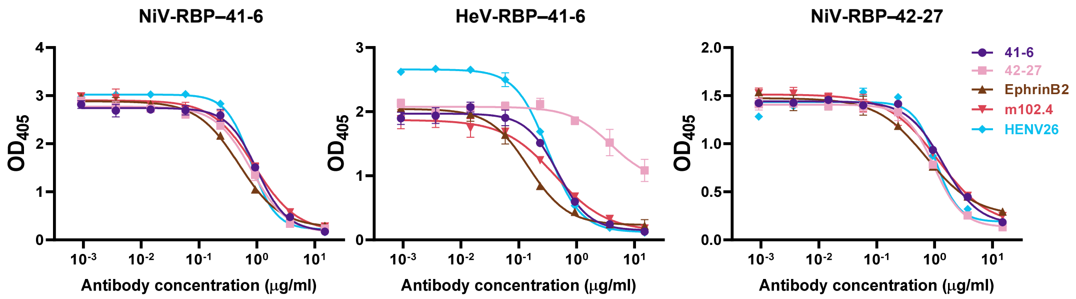

Supplementary Figure 7: Competition of receptor with different antibodies for binding to the RBPs.

RBPs were coated and incubated with 4-fold serially diluted antibodies and receptor protein in the presence of 0.03 μg/ml biotinylated 41-6 (left and middle) or 0.03 μg/ml biotinylated 42-27. The concentrations of competitive proteins were as indicated in the x axis. Data are represented as the mean ± S.D from n=3 biologically independent experiments**.** Source data are provided as a Source Data file.

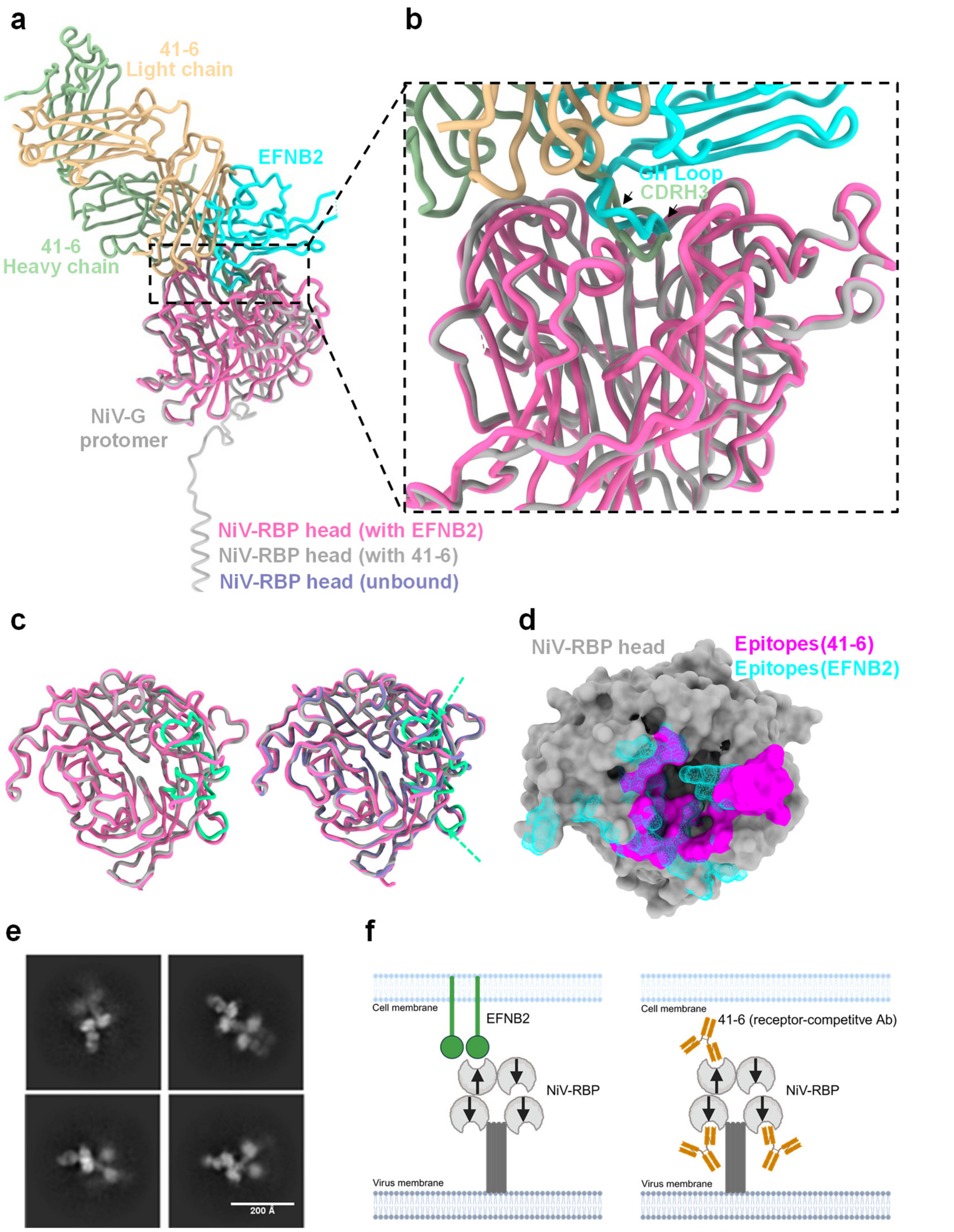

Supplementary Figure 8: Epitope comparison between antibody and receptor.

a Superimposition of NiV-RBP bound with 41-6 or with EphrinB2 (EFNB2) (PDB 2VSM) shown as ribbon diagrams (RMSD 1.06). 41-6 heavy and light chains and EFNB2 are green, tan and cyan respectively. **b** Zoomed-in view of the interface of NiV-RBP with 41-6 or EFNB2. **c** Superimposition of the NiV-RBP bound with 41-6 or EFNB2. Lime color highlights indicated regions of Cα trajectory with the deviations (3 Å >RMSD>1.5 Å), arrows indicated the changed region when bound with 41-6 not with EFNB2 or with nothing. **d** The epitope analysis of the NiV-RBP head domain superimposed bound with 41-6 or EFNB2 shown as molecular surface representation. Epitopes of NiV-RBP with

EFNB2 are highlighted in transparency cyan, magenta indicates epitopes of NiV-RBP with 41-6. **e** 2D average of complex of NiV-RBP with 41-6. **f** The model of NiV-RBP homotetramer bound with EFNB2 and receptor-like Fab fragment 41-6. Four heads structure domain is only one point to the host cell membrane receptor binding sites (arrow), while the other three sites to the virus membrane. The figure of the model was created with BioRender.com.

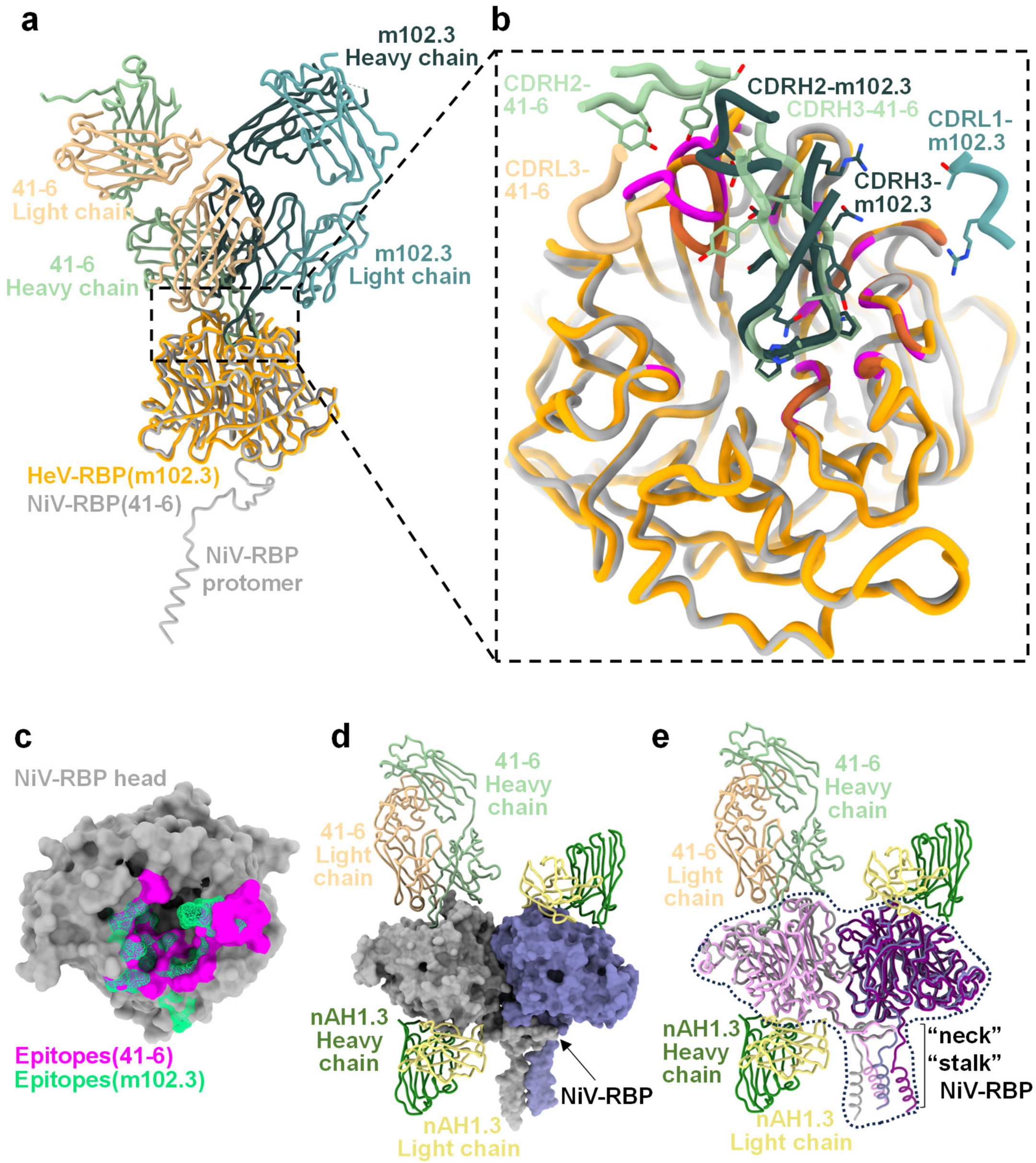

Supplementary Figure 9: Comparison of NiV-RBP with the neutralizing antibodies. **a** Superimposition of the NiV-RBP bound with 41-6 or and HeV-RBP bound with m102.3 (PDB 6CMI). HeV-RBP bound with m102.3 was fitted into NiV-RBP with 41-6 which are shown as different colored ribbon diagrams. **b** Zoomed-in view of the interface of NiV-RBP with 41-6 or m102.3, shown as ribbon diagrams. Oxygen and nitrogen atoms are colored red and blue, respectively. **c** The analysis of epitopes of the henipavirus RBP head superimposed bound with 41-6 and m102.3 shown as molecular surface representation. Epitopes of HeV-RBP with m102.3 are a transparent brown surface highlight; magenta indicates epitopes of NiV-RBP with 41-6. **d and e** Superimposition of NiV-RBP bound with 41-6 or with nAH1.3 (PDB 7TXZ). NiV-RBP highlighted by dotted lines are shown as molecular surfaces, and are gray and medium slate blue colored respectively. Fabs are shown as ribbon diagrams. **d** 41-6 and nAH1.3 bind to opposite sides of NiV-RBP head, **e** Superimposed conformation of NiV-RBP with 41-6 or nAH1.3. NiV-RBP with nAH1.3 are shown as pink and purple ribbon diagrams, respectively.

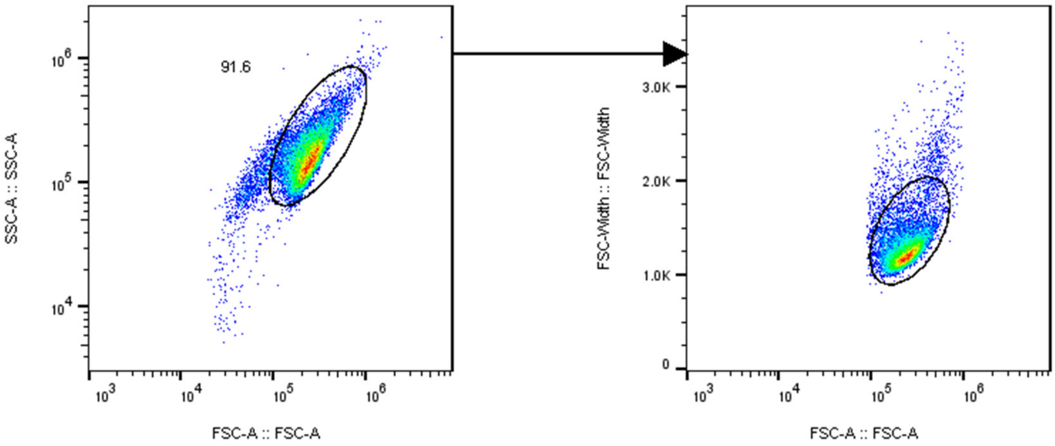

Supplementary Figure 10: Gating strategy for flow cytometry-based receptor blocking assay. Cells were gated by flow cytometry and cell aggregates. The gating strategy corresponds to the receptor blocking assay data shown in Figure 1c.

Supplementary Table 1: Summarization of the statistics of the map reconstruction and model building

	**41-6/NiV-RBP complex (EMDB-36849) (PDB 8K3C)**
**Data collection and processing**	
Magnification	105,000
Voltage (kV)	300
Electron exposure (e^-/Å^2)	51
Defocus range (μm)	-1.5~-3.2
Pixel size (Å)	0.82
Symmetry imposed	C1
Initial particle images (no.)	897,898
Final particle images (no.)	299,497
Map resolution (Å)	2.88
FSC threshold	0.143
**Refinement**	
Initial model used (PDB code)	7TXZ
Model resolution (Å)	2.9
FSC threshold	0.143
Model composition	
Non-hydrogen atoms	10813
Protein residues	1395
R.m.s. deviations	
Bond lengths (Å)	0.01
Bond angles (°)	0.03
Validation	
MolProbity score	1.95
Clashscore	8.92
Poor rotamers (%)	0.32
Ramachandran plot	
Favored (%)	92.3
Allowed (%)	7.63
Outliers/ (%)	0.07
