# Supplementary material for: Potent human neutralizing antibodies against Nipah virus derived from two ancestral antibody heavy chains
Source: Nat Commun. 2024 Apr 6;15:2987. doi: 10.1038/s41467-024-47213-8 (PMC10998907; doi:10.1038/s41467-024-47213-8)
Supplement: Supplementary file 3 — Reporting Summary [file 41467_2024_47213_MOESM3_ESM.pdf]

nature portfolio

Corresponding author(s): Rui Gong
Last updated by author(s): Mar 1, 2024

Reporting Summary

Nature Portfolio wishes to improve the reproducibility of the work that we publish. This form provides structure for consistency and transparency in reporting. For further information on Nature Portfolio policies, see our Editorial Policies and the Editorial Policy Checklist.

Statistics

For all statistical analyses, confirm that the following items are present in the figure legend, table legend, main text, or Methods section.

n/a	Confirmed	
☐	☒	The exact sample size (*n*) for each experimental group/condition, given as a discrete number and unit of measurement
☐	☒	A statement on whether measurements were taken from distinct samples or whether the same sample was measured repeatedly
☐	☒	The statistical test(s) used AND whether they are one- or two-sided<br>*Only common tests should be described solely by name; describe more complex techniques in the Methods section.*
☒	☐	A description of all covariates tested
☒	☐	A description of any assumptions or corrections, such as tests of normality and adjustment for multiple comparisons
☐	☒	A full description of the statistical parameters including central tendency (e.g. means) or other basic estimates (e.g. regression coefficient) AND variation (e.g. standard deviation) or associated estimates of uncertainty (e.g. confidence intervals)
☐	☒	For null hypothesis testing, the test statistic (e.g. *F*, *t*, *r*) with confidence intervals, effect sizes, degrees of freedom and *P* value noted<br>*Give P values as exact values whenever suitable.*
☒	☐	For Bayesian analysis, information on the choice of priors and Markov chain Monte Carlo settings
☒	☐	For hierarchical and complex designs, identification of the appropriate level for tests and full reporting of outcomes
☒	☐	Estimates of effect sizes (e.g. Cohen's *d*, Pearson's *r*), indicating how they were calculated

Our web collection on statistics for biologists contains articles on many of the points above.

Software and code

Policy information about availability of computer code

Data collection	ELISA and competitive binding assay were performed by using Gen5 CHS 2.04 (Agilent). Pseudovirus neutralization assay was performed with Operetta CLS (PerkinElmer). Biolayer interferometry was performed by using Octet RED96 (ForteBio). The germline sequence was estimated in IMGT/V-QUEST software (https://www.imgt.org/IMGT_vquest/input). Cryo-EM data was collected using EPU (version2.7). Flow cytometry-based receptor binding inhibition assay was performed using BD LSR flow cytometer.
Data analysis	GraphPad Prism 8.0.2; Octet Data Analysis 7.0; Harmony 3.5; CryoSPARC 3.2.0; MolProbity 4.5; UCSF ChimeraX 1.3; Phenix 1.19.2-4158; Coot 0.9.1.; FlowJo 10.5.3.

For manuscripts utilizing custom algorithms or software that are central to the research but not yet described in published literature, software must be made available to editors and reviewers. We strongly encourage code deposition in a community repository (e.g. GitHub). See the Nature Portfolio guidelines for submitting code & software for further information.

Data

Policy information about availability of data

All manuscripts must include a data availability statement. This statement should provide the following information, where applicable:

- Accession codes, unique identifiers, or web links for publicly available datasets
- A description of any restrictions on data availability
- For clinical datasets or third party data, please ensure that the statement adheres to our policy

The cryo-EM structures of 41-6 complexed with the NiV-RBP have been deposited in the Electron Microscopy Data Bank (EMDB) and Protein Data Bank with the

accession codes EMD- 36849, PDB ID 8K3C. This study also sued 2VSM (https://doi.org/10.2210/pdb2VSM/pdb), 3D11 (https://doi.org/10.2210/pdb3D11/pdb), 6PDL (https://doi.org/10.2210/pdb6PDL/pdb), 6CMI (https://doi.org/10.2210/pdb6CMI/pdb), 7TY0 (https://doi.org/10.2210/pdb7TY0/pdb) and 7TXZ (https://doi.org/10.2210/pdb7TXZ/pdb) from the Protein Data Bank. Other data are contained within the article/Supplementary Information. Source data are provided with this paper.

Research involving human participants, their data, or biological material

Policy information about studies with human participants or human data. See also policy information about sex, gender (identity/presentation), and sexual orientation and race, ethnicity and racism.

Reporting on sex and gender	N/A
Reporting on race, ethnicity, or other socially relevant groupings	N/A
Population characteristics	N/A
Recruitment	N/A
Ethics oversight	N/A

Note that full information on the approval of the study protocol must also be provided in the manuscript.

Field-specific reporting

Please select the one below that is the best fit for your research. If you are not sure, read the appropriate sections before making your selection.

- [x] Life sciences
- [ ] Behavioural & social sciences
- [ ] Ecological, evolutionary & environmental sciences

For a reference copy of the document with all sections, see nature.com/documents/nr-reporting-summary-flat.pdf

Life sciences study design

All studies must disclose on these points even when the disclosure is negative.

Sample size	Following standard practices, all molecular biology experiments were conducted in triplicate. In this study, sample sizes were determined according to established guidelines: a minimum of n = 3 biological independent samples for cell experiments and a minimum of n = 6 biological independent animals for animal experiments, ensuring reproducibility. Sample size were similar to those generally employed in the field (Doyle et al., 2021; Lu et al., 2023a; Lu et al., 2023b).
Data exclusions	No data was excluded.
Replication	In vitro assays were performed in 2-3 independent. In vivo protection test was performed with six hamsters per experiment group. All attempts at replication were successful.
Randomization	The allocation into experimental groups was random in all animal experiments.
Blinding	Blinding was not a relevant feature in this study. This is a non-clinical study with data collection and analyses relying on objective measures.

Reporting for specific materials, systems and methods

We require information from authors about some types of materials, experimental systems and methods used in many studies. Here, indicate whether each material, system or method listed is relevant to your study. If you are not sure if a list item applies to your research, read the appropriate section before selecting a response.

Materials & experimental systems

n/a	Involved in the study	
[ ]	[x]	Antibodies
[ ]	[x]	Eukaryotic cell lines
[x]	[ ]	Palaeontology and archaeology
[ ]	[x]	Animals and other organisms
[x]	[ ]	Clinical data
[x]	[ ]	Dual use research of concern
[x]	[ ]	Plants

Methods

n/a	Involved in the study	
[x]	[ ]	ChIP-seq
[ ]	[x]	Flow cytometry
[x]	[ ]	MRI-based neuroimaging

Antibodies

Antibodies used	1. HRP-conjugated anti-M13 mouse monoclonal antibody (HRP) (Sino Biological, cat#11973-MM05T-H, 1:3000 dilution)<br>2. A mouse monoclonal ANTI-FLAG® M2-Peroxidase (HRP) antibody (Sigma-Aldrich, cat#A8592, 1:2000 dilution)<br>3. Goat anti-human IgG Fc-DyLight 650 (Invitrogen, cat#SA5-10137, 1:50 dilution)<br>4. HRP-conjugated anti human IgG (Fc specific) (Siama-Aldrich, cat#A0170, 1:5000 dilution)<br>5. HRP-conjugated streptavidin (Siama-Aldrich, cat#18152, 1:8000 dilution)
Validation	All the secondary antibodies were bought from commercial vendors and were validated by the manufacturers<br>1. HRP-conjugated anti-M13 mouse monoclonal antibody (HRP) (https://www.sinobiological.com/antibodies/m13-11973-mm05t-h)<br>2. A mouse monoclonal ANTI-FLAG® M2-Peroxidase (HRP) antibody (https://www.sigmaaldrich.cn/CN/en/product/sigma/a8592)<br>3. Goat anti-human IgG Fc-DyLight 650 (https://www.thermofisher.cn/cn/zh/antibody/product/Goat-anti-Human-IgG-Fc-Cross-Adsorbed-Secondary-Antibody-Polyclonal/SA5-10137)<br>4. HRP-conjugated anti human IgG (Fc specific) (https://www.sigmaaldrich.cn/CN/en/product/sigma/A0170)<br>5. HRP-conjugated streptavidin (https://www.sigmaaldrich.cn/CN/en/product/mm/18152

Eukaryotic cell lines

Policy information about cell lines and Sex and Gender in Research

Cell line source(s)	Vero cells (catalog no. GDC0029), Vero E6 cells (catalog no. GDC0146) and 293T cells (catalog no. GDC0187) were obtained from the China Center for Type Culture Collection (CCTCC). FreeStyleTM 293-F cells (cat#R79007) were obtained from ThermoFisher
Authentication	All cell lines were previously reported but not authenticated by us.
Mycoplasma contamination	All used cell stocks tested negative for mycoplasma.
Commonly misidentified lines (See ICLAC register)	No commonly misidentified cell lines were used.

Animals and other research organisms

Policy information about studies involving animals; ARRIVE guidelines recommended for reporting animal research, and Sex and Gender in Research

Laboratory animals	Five- to six-week-old female Syrian golden hamsters were randomly allocated into groups. They were kept in SPF animal facilities at the Wuhan Institute of Virology, Chinese Academy of Sciences. The relative humidity was kept at 45 to 65%. Animal rooms and cages were kept at a temperature range of 20 to 24°C, and were set at a 12:12 for the light:dark cycle. Viral infection was conducted in a BSL-4 facility in accordance with the guidelines for the care and use of laboratory animals and the Institutional Review Board of the Wuhan Institute of Virology, Chinese Academy of Sciences.
Wild animals	No wild animals were used in the study.
Reporting on sex	Only female mice were used in this study, as male mice are more aggressive than female mice. Subsequent studies in Nipah virus pathogenicity in hamsters showed no variability between the sexes.
Field-collected samples	No field-collected samples were used in the study.
Ethics oversight	Hamster studies were approved by the Life Science Ethics Committee of the Wuhan Institute of Virology , Chinese Academy of Sciences. (approval no. WIVA45202306).

Note that full information on the approval of the study protocol must also be provided in the manuscript.

Plants

Seed stocks	N/A
Novel plant genotypes	N/A
Authentication	N/A

Flow Cytometry

Plots

Confirm that:

- [x] The axis labels state the marker and fluorochrome used (e.g. CD4-FITC).
- [x] The axis scales are clearly visible. Include numbers along axes only for bottom left plot of group (a 'group' is an analysis of identical markers).
- [x] All plots are contour plots with outliers or pseudocolor plots.
- [x] A numerical value for number of cells or percentage (with statistics) is provided.

Methodology

Sample preparation	Vero cells grown to subconfluency were detached by sodium citrate and aliquoted into tubes. After washing with PBS+2% FBS, the cells were blocked with 1% BSA in PBS on ice. Tenfold serial dilutions of Fabs were incubated with NiV-RBP-Fc (at a final concentration of 10 nM) on ice for 1 hour, and then the mixture was added to cells for incubation on ice for 1.5 hours. Cells incubated with RBP-Fc were used as a positive control. After washing three times with PBS+2% FBS, the cells were stained with goat anti-human IgG Fc-DyLight 650 on ice for 1 hour. Following three times washes, the cells were resuspended and analyzed by flow cytometry using a BD LSR flow cytometer (BD Biosciences).
Instrument	Flow cytometry were performed using BD LSR flow cytometer.
Software	Data were analyzed by FlowJo 10.5.3.
Cell population abundance	All cells are of the same type; therefore, the concept of cell population abundance is not relevant in this study.
Gating strategy	Cells were gated by FSC/SSC. Cell aggregates were excluded by FSC-A/FSC-W.

- [x] Tick this box to confirm that a figure exemplifying the gating strategy is provided in the Supplementary Information.
